# Effect of collaborative nursing method based on RAM model on postoperative functional reconstruction, soft tissue pain and living quality in patients with femoral trochanter fracture

**DOI:** 10.1186/s12891-024-07746-x

**Published:** 2024-08-06

**Authors:** Xiaoqing Shi, Wencan Ma

**Affiliations:** 1 Department of Trauma, No.4 Trauma area. Hangzhou Fuyang Hospital of TCM Orthopedics and Traumatology, Hangzhou City, Zhejiang Province China; 2 Department of Radiology, Hangzhou Fuyang Hospital of TCM Orthopedics and Traumatology, Zhejiang Province No. 418, Fengpu Road, Fuchun Street, Fuyang District, Hangzhou City, China

**Keywords:** RAM model, Collaborative nursing, Femoral intertrochanteric fracture, Functional reconstruction, Soft tissue pain, Quality of life

## Abstract

**Objective:**

To explore the effect of collaborative nursing based on Roy Adaptive Mode (RAM) on postoperative functional reconstruction, soft tissue pain and quality of life in patients with femoral intertrochanteric fracture.

**Methods:**

A retrospective matched control method was used in this study. A total of 96 patients with femoral intertrochanteric fracture admitted to our hospital from July 2018 to September 2021 were selected. According to different nursing methods, the patients were divided into a collaborative group and a routine group, with 48 cases in each group. Patients in both groups were treated with intramedullary nail surgery. The routine group was given routine perioperative nursing intervention, and the collaborative group was given collaborative nursing intervention on this basis. The hip function recovery and quality of life before and after the intervention were compared between the two groups. The preoperative and postoperative pain degree, and the perioperative complications of the two groups were recorded. Logistic multivariate regression analysis was used to analyze the risk factors affecting the recovery of hip joint function in patients with femoral intertrochanteric fracture after operation, thereby constructing a risk prediction model. ROC curve was used to analyze the clinical value of influencing factors in predicting postoperative hip function recovery in patients with femoral intertrochanteric fracture.

**Results:**

Harris score each dimension after intervention in the collaborative group was obviously higher than that of before intervention and the conventional group (*P* < 0.05). After intervention, the excellent and good rate of hip joint function the collaborative group was 83.33%, which was significantly higher than 60.42% in the routine group (*P* < 0.05). Postoperative VAS scores each time point in the collaborative group was obviously lower than that in the routine group (*P* < 0.05). After intervention, the scores of physiological function, physiological role, body pain and general health in the collaborative group were significantly higher than those in the routine group (*P* < 0.05). The incidence of complications in the collaborative group was 6.25%, which was significantly lower than 22.92% in the routine group (*P* < 0.05). There were statistically significant differences in age, preoperative ASA grade, internal fixation method, osteoporosis grade and perioperative nursing methods between the excellent hip recovery group and the poor hip recovery group (*P* < 0.05). Logistic multivariate regression analysis showed that age, preoperative ASA grade, internal fixation method and osteoporosis grade were the risk factors affecting the recovery of hip joint function after operation, and perioperative nursing method was the protective factor (*P* < 0.05). Among the influencing factors, the internal fixation method and the grade of osteoporosis had certain clinical value in predicting the recovery of hip joint function in patients with femoral intertrochanteric fracture after operation.

**Conclusion:**

The RAM model-based collaborative nursing method may effectively restore the hip joint function of patients with femoral intertrochanteric fracture after operation, and may reduce the perioperative pain degree of patients, improve the quality of life of patients and reduce the incidence of complications, which can be popularized and applied in clinical practice. In addition, there are many factors influencing the recovery of hip joint function in patients with femoral intertrochanteric fracture after operation, and targeted measures should be taken according to the influencing factors to improve the effect of intramedullary nail treatment.

## Introduction

Femoral intertrochanteric fracture refers to the fracture at the base of the femoral neck and above the level of the lesser trochanter. It accounts for about 3.12% of all fractures, and the elderly are the main population. The incidence of fracture is basically the same as that of femoral neck fractures. However, because of the rich blood supply in the intertrochanteric region, fracture nonunion is rare, but it is prone to coxa vara, especially for elderly patients with poor physical conditions [1, 2]. At present, early surgical treatment is advocated for femoral trochanteric fracture in the elderly. Intramedullary nail surgery is the best therapy, which can provide strong internal fixation for the fracture end and is conducive to early fracture healing of the fracture.

The skeletal tissue of elderly patients usually undergoes significant degradation, bone microstructure damage, increased brittleness, and poor tolerance to anesthesia and surgery, thus the patients often have negative emotions, such as anxiety and tension, which are not conducive to the implementation of surgery and the postoperative fractures healing. At this time, the implementation of collaborative nursing intervention in the perioperative period of patients with femoral trochanter fracture can effectively reduce the influence of the these factors on postoperative recovery. Collaborative nursing intervention is a nursing intervention carried out by multidisciplinary collaboration on the basis of responsible nursing, which mainly emphasizes the subjective initiative of patients, cultivates the self-care ability of patients, and reduces the pressure and burden of the caregiver [3, 4]. Roy Adaptation Model (RAM) is a kind of nursing theory, widely applied in the field of nursing at home and abroad. This model thoroughly explores human adaptation mechanisms, methods and processes, and combines adaptation models with general nursing models to assist patients in the face of stimulation can enhance their self-concept, characters, physiological and social function of adaptation, to promote the recovery of the patients [5]. RAM believes that a person is an organic whole with biological, psychological and social attributes, which regards the whole life process of a person as a process of self-adjustment and adaptation of the body. It is a holistic comfort nursing model that is consistent with the modern medical model. During the development of the adaptation model, Roy endows concepts from outside the nursing field with new connotations and creatively integrate them into the nursing field [6]. From the holistic view, it focuses on the coping mechanism, adaptive mode and adaptive process of human being as an adaptive system in the face of various stimuli in the internal and external environment. To enhance effective adaptation, the nurse should lose no time to adaptation problem of the individual or group as well as the problems caused by stimulus to judge and intervention, so as to strengthen the ability to adapt and improve adaptive reaction, improve the level of health [7].

This study explored the impact of the RAM model-based collaborative nursing method on postoperative functional reconstruction, soft tissue pain and quality of life in patients with femoral trochanter fractures.

## Materials and methods

### General data

**Fig. 1:**
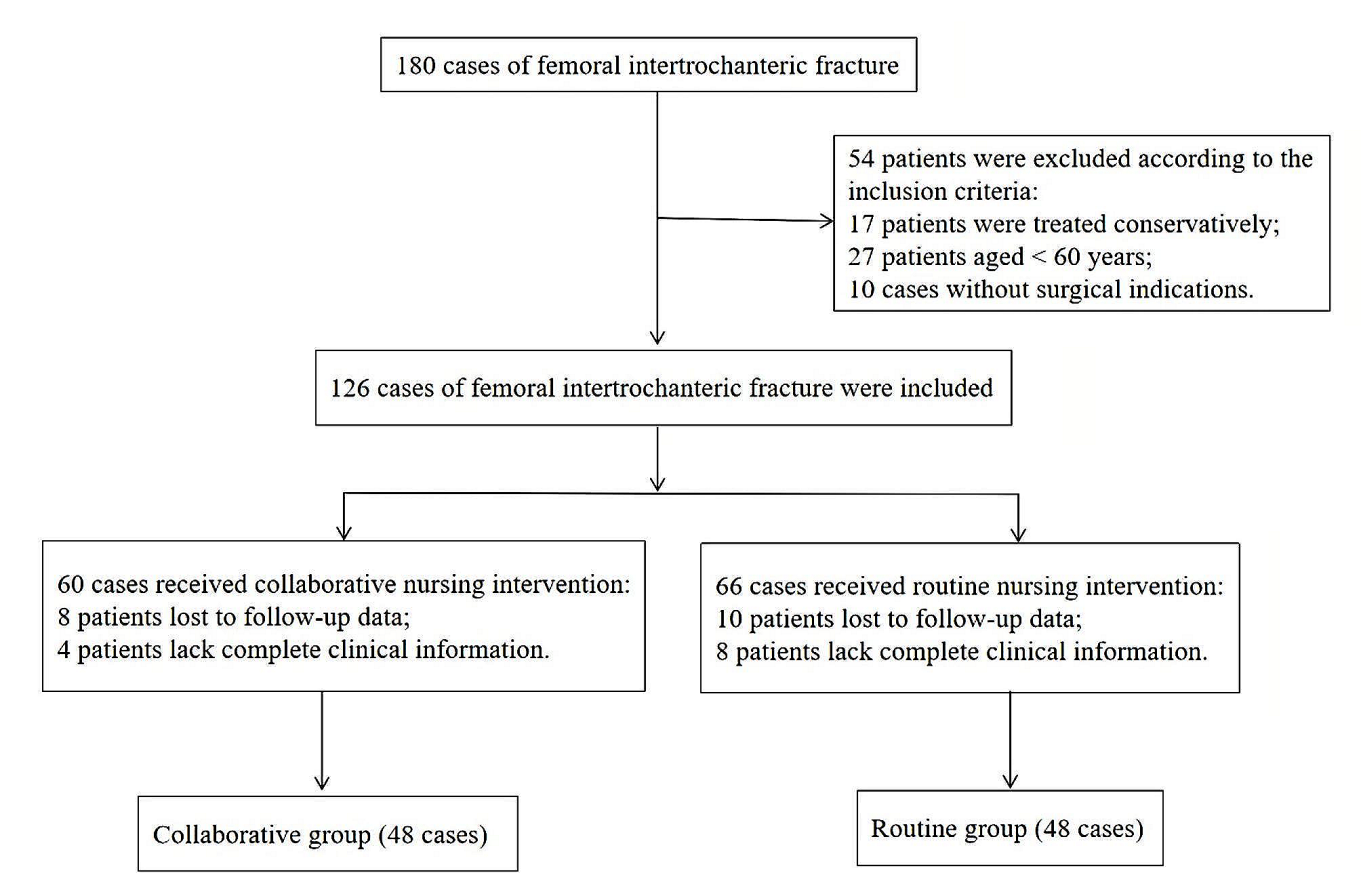
Flow chart of general information selection

A retrospective matched control method was used in this study. A total of 96 patients with femoral intertrochanteric fracture admitted to our hospital from July 2018 to September 2021 were selected as the research objects (Fig. 1). This study was approved by the ethics committee of our hospital. Inclusion criteria: (1) Patients treated with intramedullary nail surgery; (2) Patients with greater trochanter fractures diagnosed by orthopedic examination and imaging examination; (3) Age ≥ 60 years old, and fracture time ≤ 2 weeks; (4) Patients with clear mind, normal thinking, and able to communicate verbally; (5) Informed consent was expressed in writing by the patient or, if not available, signed by a relatives or tutors. Exclusion criteria: (1) Patients with injury in liver and kidney and other important organ dysfunction; (2) Patients with other fracture types or other orthopedic diseases; (3) Patients with spinal cord nerve injury; (4) Patients with preoperative severe infection and pressure ulcers; (5) Patients with spirit, consciousness, communication disorders of patients; (6) Patients with physical intolerance and other surgical contraindications. According to the different nursing methods, the patients were divided into the collaborative group and the routine group. Patients in the routine group who underwent surgery from July 2018 to December 2019 were treated with orthopedic routine nursing, and patients in the collaborative group who underwent surgery from January 2020 to September 2021 were treated with collaborative nursing based on RAM mode, with 48 cases in each group. There was no difference in general data between the two groups (*P* > 0.05, Table 1).

**Table 1:** Comparison of general data ($\bar x \pm s$), [n (%)]

General data		Collaborative group (*n* = 48)	Routine group (*n* = 48)	χ^2^/t	*P*
Gender	Male	25 (52.08)	29 (60.42)	0.677	0.411
	Female	23 (47.92)	19 (39.58)		
Age (years)		70.25 ± 11.48	72.16 ± 11.68	0.808	0.421
Educational degree	Primary and below	11 (22.92)	9 (18.75)	0.676	0.713
	Junior high school	15 (31.25)	13 (27.08)		
	High school and above	22 (45.83)	26 (54.17)		
Fracture site	Left	28 (58.33)	25 (52.08)	0.379	0.538
	Right	20 (41.67)	23 (47.92)		
Cause of injury	Traffic accident	13 (27.08)	16 (33.33)	0.794	0.851
	Fall injury	20 (41.67)	17 (35.42)		
	Fall from height	12 (25.00)	13 (27.08)		
	Others	3 (6.25)	2 (4.17)		

### Nursing methods

Patients in the routine group and the collaborative group were treated with intramedullary nailing surgery. On the basis of surgical treatment, patients in the two groups were given routine perioperative nursing measures, including observation nursing, guiding of regular diet, prevention and treatment of complications, medication guidance and rehabilitation guidance. The patients in the collaborative group were given with collaborative nursing intervention on this basis, and the specific measures were as follows:

According to the problems encountered in clinical nursing practice of orthopedic patients during hospitalization, on the basis of reading and analyzing a large number of literatures, combined with clinical practice and Roy adaptation model, the patients were evaluated from four aspects of physiological function, self-concept, role function and interdependence to find out the ineffective response of patients. The main stimuli, related stimuli and intrinsic stimuli that caused ineffective responses were identified, and the nursing problems were initially established. Demonstrating the RAM model-based collaborative nursing intervention program: on-site expert argument-peer review. The first draft of the protocol was consulted by joint surgical clinicians and nursing experts, and the consensus was reached. The final draft of collaborative nursing intervention plan based on RAM model was formed. The first draft of the plan was revised in combination with the results of pre-trial investigation and on-site expert demonstration, and finally a perfect collaborative nursing intervention plan was formed.

1. Establishment of the collaborative nursing model team: The team members included orthopedic surgeons, rehabilitation doctors, psychological consultants, head nurses and responsible nurses. Group training was conducted before the study, including the content, methods, and skills of collaborative nursing models. All of them passed the assessment and had the ability to deal with nursing emergencies independently.
2. Health education: Doctors, nurses and rehabilitation therapists jointly carried out preoperative education for family members and patients, distributed perioperative nursing manuals for elderly patients with femoral intertrochanteric fracture, and explained the purpose and method of operation and the coping style of intraoperative accidents to patients through simple language or graphics according to patients’ acceptance and understanding ability, so as to increase the patient’s confidence in anesthesia and surgery. At the same time, the patients were explained by the importance of following the doctor’s recommendations for disease recovery.
3. Psychological nursing intervention: Femoral tuberosity fractures were more common in the elderly, and these patients were relatively sensitive psychologically. Faced with bed rest and inability to take care of themselves caused by sudden fracture, they are easy to show anxiety, tension and depression and other bad psychological conditions. Nursing staff should actively communicate with patients and their families, timely understand the psychological needs of patients, patiently listen to the aspirations of patients, and increase the psychological sense of security of patients. Nursing staff should explain the necessity of surgery treatment, introduce the successful cases, guide the patients correctly treat disease, and increase the confidence. Nursing staff can let patients listen to soothing music and watch TV, guide patients to actively divert attention and maintain a comfortable state of mind.
4. Postoperative care: The patient was placed in the lateral position within 24 h after operation, and the patient was guided to turn over the axis to avoid spinal distortion. The patient’s pain characteristics were observed and recorded after the anesthesia subsides. Visual analogue scale method was used to evaluate patients’ pain every 4 h. Encourage patients to express the pain, to accept the feelings of pain and reaction. The patient was distracted from pain by teaching self-suggestion, relaxation techniques, music therapy, and distraction. The patients were instructed to avoid large movements in the early postoperative period and to avoid pain caused by pulling the wound. For patients with score of 7 points, hot or cold compresses were appropriately performed on the incision to alleviate the pain, with the use of analgesic drugs in accordance with the doctor’s advice.
5. Rehabilitation nursing: The rehabilitation exercise was carried out one to one by the rehabilitator, and isometric contraction training of quadriceps femoris was carried out on the second day after operation. Three to five days after operation, the patients were guided to perform knee joint and hip joint activities on the bed and bedside. One week after surgery, the patients were instructed to get out of bed, wear waist circumference and walk with the help of crutching stick or wall. Hip flexion exercise was performed according to the patient’s condition at 3 weeks after operation.
6. Complication prevention: The nursing staff made rounds every 2 h after operation, guided the patients and their families to carry out limb massage according to the changes of the patient’s signs and the condition of the incision, so as to prevent the occurrence of complications such as deep vein thrombosis and pressure ulcers. Actively assist patients with sputum, and keep respiratory tract clear. Guide patients urinate regularly, and prevent urinary tract infection.
7. Diet guidance: Make scientific diet plan, guide the patients to eat more food that was easy to digest, high vitamin, rich in protein and calcium food, and to eat fresh fruit, and prevent constipation.

### Observation indexes

1. Harris score about hip joint function: Harris score system was used to assess the recovery of hip joint function of patients in the two groups before intervention and 3 months after the intervention. The scale included 7 items, including pain degree, daily activity function, range of motion, gait, walking aid, lower limb deformity, and walking distance. The total score was 100 points, and the score was proportional to the hip joint function. According to Harris score, the hip joint function was graded as excellent (90–100 points), good (80–89 points), moderate (70–79 points) and poor (< 70 points). Excellent and good rate = (excellent + good) / total cases × 100%.
2. Pain score: The visual analogue score (VAS) was used to assess the patients’ pain in the preoperative and postoperative 6 h, 12 h, and 24 h. Score was from 0 to 10. The higher the score was, the more severe the pain was.
3. Living quality: The Short form 36 (SF-36) [8] was used to evaluate the quality of life of patients in the two groups before and 3 months after the intervention. The scale included 8 dimensions, the score was 0-100, and the score was proportional to the quality of life.
4. Complications: The perioperative complications were recorded in the patients between the two groups.
5. The data of gender, age, preoperative ASA classification, time from injury to operation, internal fixation methods [Proximal Femoral Anti-rotation Intramedullary Nail (PFNA), Hollow Nail, Dynamic Hip Screw (DHS)], and osteoporosis classification [9] were collected.

### Statistical methods

SPSS20.0 software package was used for analysis of data in this research. Measurement data were expressed as ($\bar x \pm s$ ), and independent sample T was used to analyze the data of collaborative group and routine group. Enumeration data were expressed by [n (%)]. *χ*^*2*^ test was used to compare the data between the two groups, and Logistic multifactor regression was used to analyze the risk factors influencing the functional recovery of the hip joint in patients with femoral intertrochanteric fracture, so as to construct a risk prediction model. The ROC curve predicted the clinical value of postoperative hip function recovery in patients with femoral intertrochanteric fracture. *P* < 0.05 was regarded as statistically significant difference.

## Results

### Comparison in Harris scores of the hip joint function between the two groups

No significant difference in Harris scores of the hip joint function was observed between the two groups before intervention (*P* > 0.05, Table 2). Harris scores of the hip joint function in the two groups after the intervention were higher than those before the intervention (*P* < 0.05), and Harris scores in the collaborative group were higher than those in the routine group after the intervention (*P* < 0.05, Table 2).

**Table 2:** Comparison of Harris score of hip function between the two groups ($\bar x \pm s$)

Item	Collaborative group (*n* = 48)		Routine group (*n* = 48)	
	Before intervention	After intervention	Before intervention	After intervention
Pain degree	7.59 ± 9.24	40.16 ± 5.25*^#^	7.56 ± 8.47	37.59 ± 5.16*
Daily activity function	2.29 ± 2.25	11.96 ± 1.54*^#^	2.11 ± 3.15	8.22 ± 1.46*
Gait	1.69 ± 2.44	10.26 ± 1.37*^#^	1.82 ± 2.34	8.39 ± 2.05*
Walking aid	1.52 ± 2.16	9.42 ± 1.59*^#^	1.59 ± 2.37	6.74 ± 2.16*
Walking distance	1.92 ± 2.27	8.55 ± 1.78*^#^	2.01 ± 2.84	7.25 ± 2.12*
Lower limb malformation	2.39 ± 0.74	3.59 ± 0.42*^#^	2.25 ± 0.46	2.88 ± 0.59*
Range of activity	2.46 ± 0.58	3.29 ± 0.56*^#^	2.16 ± 0.85	2.69 ± 0.54*
Total points	19.78 ± 17.56	87.05 ± 6.44*^#^	19.62 ± 18.67	74.46 ± 8.02*

Note: Compared with the same group before intervention, *P < 0.05; Compared with the routine group after intervention, #*P* < 0.05

### Comparison of hip function grades

The excellent and good rate of hip function in the collaborative group was 83.33% after intervention, which was higher than the 60.42% of the routine group (*P* < 0.05, Table 3).

**Table 3:** Comparison of hip function grades between the two groups [n (%)]

Groups	Cases	Excellent	Good	Medium	Low	Excellent and good rate
Collaborative group	48	13 (27.08)	27 (56.25)	8 (16.67)	0 (0.00)	40 (83.33)
Routine group	48	7 (14.58)	22 (45.83)	11 (22.92)	8 (16.67)	29 (60.42)
*χ* ^*2*^						6.235
*P*						0.013

### Comparison of perioperative pain scores

There was no significant difference in VAS score between the two groups before intervention (*P* > 0.05, Table 4). The VAS score in the collaborative group was lower than that in the routine group at each time point after intervention (*P* < 0.05, Table 4).

**Table 4:** Comparison of perioperative pain scores between the two groups ($\bar x \pm s$)

Groups	Cases	Preoperative	6 h after operation	12 h after operation	24 h after operation
Collaborative group	48	7.15 ± 1.33	4.16 ± 1.26*	3.18 ± 1.02*	2.62 ± 0.73*
Routine group	48	7.29 ± 1.41	5.01 ± 1.15*	3.90 ± 1.11*	3.48 ± 1.11*
*t*		0.500	3.452	3.309	4.485
*P*		0.618	0.001	0.001	< 0.001

Note: Compared with the same group before operation, *P < 0.05

### Comparison of scores in quality of life between the two groups

After intervention, the physiological function, physiological function, physical pain, and overall health scores of patients in the collaborative group were better than those in the routine group (*P* < 0.05, Table 5).

**Table 5:** Comparison of scores in quality of life between the two groups ($\bar x \pm s$)

Item	Collaborative group (*n* = 48)		Routine group (*n* = 48)	
	Before intervention	After intervention	Before intervention	After intervention
Physiological function	29.8 ± 9.42	65.24 ± 6.41*^#^	48.25 ± 5.40	52.25 ± 8.74*
Role-physieal	63.55 ± 3.74	70.58 ± 8.16*^#^	60.25 ± 5.44	64.12 ± 8.56*
Body pain	75.45 ± 5.53	85.52 ± 5.05*^#^	74.85 ± 8.51	78.12 ± 8.23*
General health	74.12 ± 3.51	86.70 ± 3.52*^#^	75.59 ± 3.01	77.51 ± 4.06*
Energy	55.25 ± 5.12	60.44 ± 4.05*	57.55 ± 4.16	60.26 ± 3.34*
Social function	55.14 ± 3.01	57.85 ± 2.71*	57.01 ± 3.53	59.35 ± 3.26*
Emotional function	74.26 ± 6.48	76.25 ± 2.85*	75.45 ± 2.48	77.82 ± 3.74*
Mental Health	67.52 ± 5.13	71.16 ± 4.26*	70.16 ± 2.14	71.66 ± 3.87

Note: Compared with the same group before intervention, *P < 0.05; Compared with the routine group after intervention, #*P* < 0.05

### Comparison of complications between the two groups

The incidence of complication of patients in the collaborative group was 6.25%, which was lower than the 22.92% in the routine group (*P* < 0.05, Table 6).

**Table 6:** Comparison of complications between the two groups [n (%)]

Groups	Cases	Pressure sores	Fracture displacement	Deep vein thrombosis	Constipation	Total incidence
Collaborative group	48	1 (2.08)	0 (0.00)	0 (0.00)	2 (4.17)	3 (6.25)
Routine group	48	3 (6.25)	4 (8.33)	2 (4.17)	2 (4.17)	11 (22.92)
*χ* ^*2*^						5.352
*P*						0.021

### Univariate analysis of affecting factors of hip joint recovery in patients with femoral trochanter fracture

The results of univariate analysis showed that there were statistically significant differences in age, preoperative ASA classification, internal fixation methods, osteoporosis classification, and perioperative nursing methods of patients with femoral intertrochanteric fractures in the excellent hip recovery group and the poor hip recovery group (*P* < 0.05, Table 7).

**Table 7:** Univariate analysis of influencing factors of hip joint recovery in patients with femoral trochanter fracture

		Cases	Excellent and good group (*n* = 69)		Medium and low group (*n* = 27)	χ^2^	*P*
Gender	Male	54	39 (56.52)	15 (55.56)		0.007	0.932
	Female	42	30 (43.48)	12 (44.44)			
Age	60 ~ 69 years	46	37 (53.62)	9 (33.33)		11.974	0.003
	70 ~ 79 years	32	25 (36.23)	7 (25.93)			
	≥ 80 years	18	7 (10.14)	11 (40.74)			
Preoperative ASA classification	I	30	26 (37.68)	4 (14.81)		11.983	0.002
	II	58	41 (59.42)	17 (62.96)			
	III	7	2 (2.90)	6 (22.22)			
Injury until operation time	< 7d	60	43 (62.32)	17 (62.96)		0.226	0.893
	7 ~ 14d	23	16 (23.19)	7 (25.93)			
	≥ 14d	13	10 (14.49)	3 (11.11)			
Internal fixation method	PFNA	71	57 (82.61)	14 (51.85)		13.706	0.001
	Hollow rivets	14	9 (13.04)	5 (18.52)			
	DHS	11	3 (4.35)	8 (29.63)			
Osteoporosis grading	Level 1 ~ 3	74	61 (88.41)	13 (48.15)		17.804	< 0.001
	Level 4 ~ 6	22	8 (11.59)	14 (51.85)			
Perioperative nursing methods	Collaborative nursing	48	40 (57.97)	8 (29.63)		6.235	0.013
	Routine nursing	48	29 (42.03)	19 (70.37)			

### Logistic multivariate regression analysis of age

**Fig. 2:**
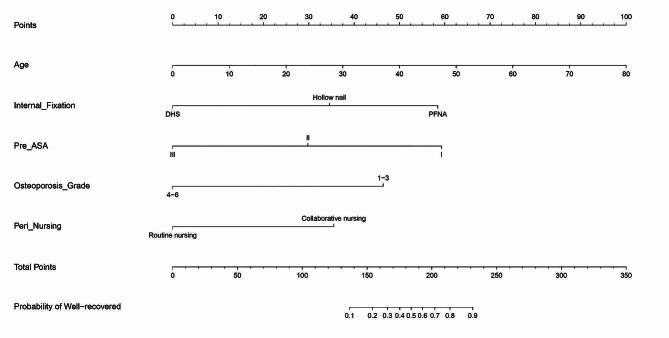
Nomogram analysis

The factors with statistical significance in Table 7 were used as independent variables, including age (0 = 60–79 years old, 1 = ≥ 80 years old), preoperative ASA classification (0 = grade I, II, 1 = grade III), internal fixation method (0 = PFNA, cannulate nail, 1 = DHS), osteoporosis classification (0 = grade 1–3, 1 = grade 4–6), and perioperative nursing methods (0 = collaborative nursing, 1 = routine nursing), and the postoperative hip function recovery was used as the dependent variable (0 = excellent, 1 = poor) for Logistic multivariate regression analysis. The results showed that age, preoperative ASA classification, internal fixation method, and osteoporosis classification were risk factors affecting the restore of hip joint function post-operation, and perioperative nursing methods were protective factors (*P* < 0.05, Table 8). The nomogram results showed that patients with younger age, lower ASA grade, PFNA internal fixation, lower osteoporosis grade and collaborative nursing intervention had good postoperative hip function recovery (Fig. 2).

**Table 8:** Logistic multifactor regression analysis

Factors	B	SE	Wald	*P*	OR	95% CI
Age	0.058	0.020	8.408	0.004	1.060	1.019 ~ 1.102
Preoperative ASA classification	2.741	1.232	4.948	0.026	15.501	1.385 ~ 173.463
Internal fixation method	2.704	0.992	7.429	0.006	14.932	2.137 ~ 104.331
Osteoporosis grading	2.147	0.710	9.135	0.003	8.555	2.127 ~ 34.416
Perioperative nursing methods	-2.310	1.034	4.979	0.015	0.099	0.012 ~ 0.758

### ROC curve analysis

**Fig. 3:**
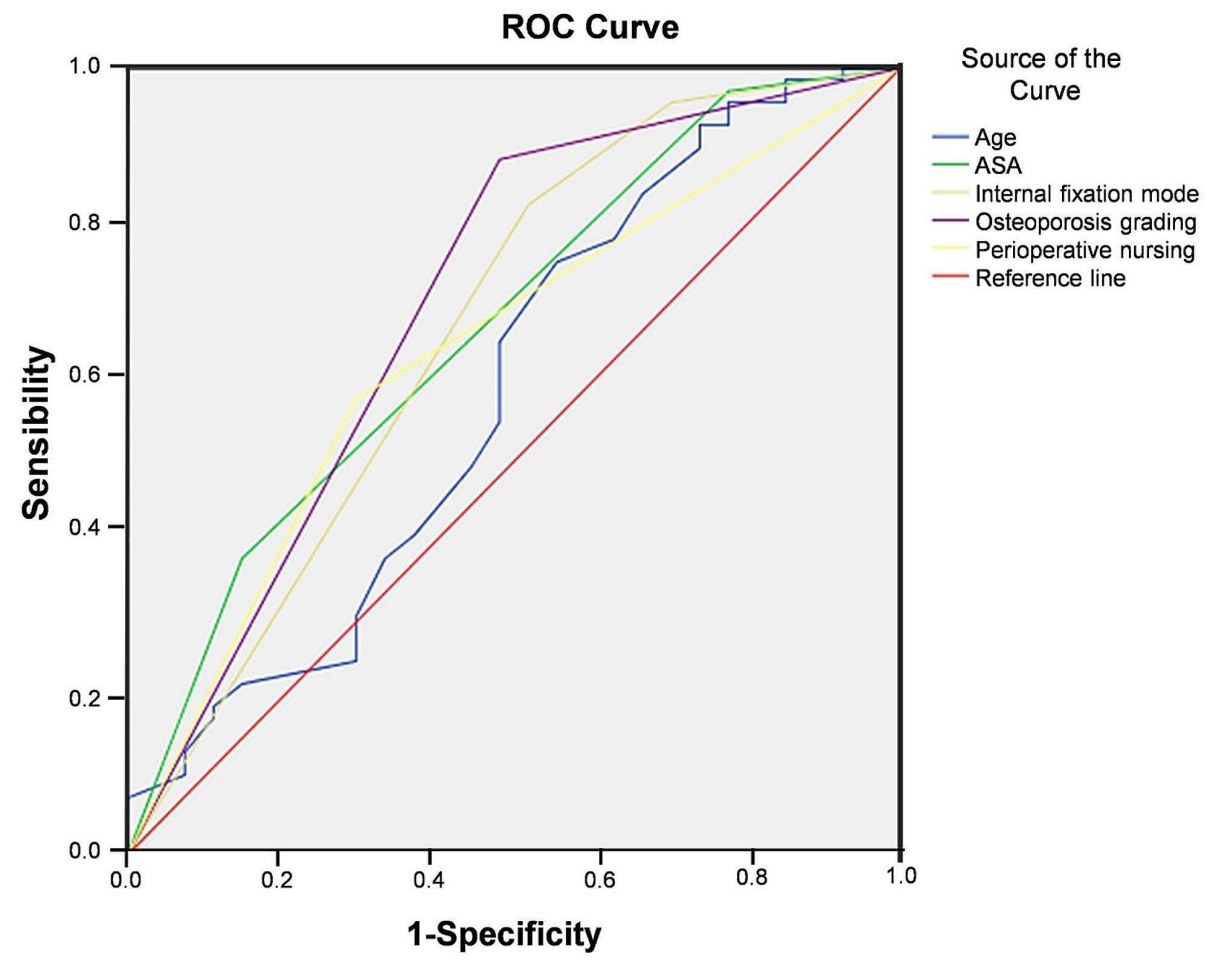
ROC curve analysis diagram

The recovery of hip joint function after operation was used as the dependent variable (0 = excellent, 1 = poor), and the influencing factors were used as independent variables for ROC curve analysis. The results showed that internal fixation methods and osteoporosis grading had certain clinical value in predicting the recovery of hip joint function after operation in patients with femoral intertrochanteric fracture (Table 9, and Fig. 3).

**Table 9:** ROC curve analysis

Factors	AUC	SE	*P*	95% CI	Sensibility	Specificity
Age	0.589	0.069	0.175	0.454 ~ 0.725	61.50	78.30
Preoperative ASA classification	0.671	0.062	0.009	0.550 ~ 0.792	50.00	97.10
Internal fixation method	0.669	0.067	0.010	0.538 ~ 0.800	82.65	64.22
Osteoporosis grading	0.701	0.065	0.002	0.574 ~ 0.828	91.62	65.78
Perioperative nursing methods	0.642	0.062	0.031	0.520 ~ 0.763	62.35	64.58

## Discussion

The incidence of femoral intertrochanteric fractures is high in the elderly. Proximal femoral anti-rotation intramedullary nail fixation is still the main clinical treatment measure, which can effectively restore the bone anatomical structure, promote the recovery of limb function, and greatly reduce the disability rate. Elderly patients have many underlying diseases and poor postoperative healing ability, which have adverse effects on the recovery of joint function. The quality of postoperative nursing is directly related to the rehabilitation process and prognosis of patients. Therefore, effective nursing methods are of great significance to reduce the pain and improve the quality of life of patients [10]. Elderly patients with femoral intertrochanteric fracture have the particularity. The traditional way of nursing is passive mechanization and easy to ignore the influence the outcome and rehabilitation of disease on the details of the problem, which cannot fundamentally improve the quality of nursing, and is difficult to meet the demand of postoperative femoral intertrochanteric fracture patients.

The collaborative nursing intervention based on the RAM model is a kind of people around the adaptive behavior and normative nursing means. It treats people as a whole adaptive system. Under the precondition of mastering the true situation of patients, it implements a variety of targeted nursing interventions. The intervention promotes patients’ adaptability to physiological functions, role functions, interdependence, and self-concept, which helps patients to treat diseases actively and correctly, and promotes patients to adapt to changes in their own roles more quickly [11]. Collaborative nursing intervention has been widely used in primary care or community medical institutions abroad. The studies has found that collaborative care can maximize the self-confidence and enthusiasm of patients and their families, pay attention to the collaboration between multidisciplinary personnel, so as to improve the self-care ability and compliance of patients, effectively and comprehensively give patients physical, psychological and social expenditure, and reduce the burden of caregivers and society [12, 13]. Results from this study showed that Harris scores of all dimensions in the collaborative group were larger than those in the routine group, and the excellent and good rate of the hip function in the collaborative group was 83.33%, which was higher than the 60.42% of the routine group. It can be seen that this nursing model can effectively promote the recovery of hip joint function in patients with femoral intertrochanteric fractures after surgery compared with routine nursing. Analysis of the relevant reasons may be that patients and their families in the collaborative group received disease-related knowledge guidance and psychological counseling before operation, which fully mobilized the enthusiasm of patients. Besides, postoperative pain nursing could promote the follow-up rehabilitation exercise or treatment compliance of patients, and strengthening the propaganda and education of patients’ families was convenient for patients to get timely guidance after discharge, so as to help to improve the postoperative patients with hip joint function. After intervention, the scores of physiological function, physiological function, physical pain, and overall health of the patients in the collaborative group were higher than those in the routine group. In the collaborative group, family members were encouraged to actively contact patients with the outside world, which could make patients subjectively and objectively get more social support, maintain the realization of self-worth and happy mood, and help patients actively and optimists to integrate into social life. However, the quality of social life of patients in the collaborative group was still at a moderate level after intervention, which might be related to the limited intervention time of this study. These results suggested that nursing staff should pay more attention to the psychological status, self-concept, role function, and interdependence of patients to diagnose and manage ineffective responses to the RAM model. The RAM model theory as the theoretical framework was used to guide nursing staff at the femoral intertrochanteric fracture patient care from the above aspects, which can analyze and identify patients facing all kinds of stimulation, make nursing diagnosis, set goals of nursing, implement effective to patients with personalized nursing intervention to promote overall adaptation, reduce the psychological burden of patients, make patients actively cooperate with the treatment and nursing, so as to improve the clinical curative effect of diagnosis and treatment and the quality of life of patients [14]. In addition, the results of this study found that the complication rate of patients in the cooperative group was 6.25%, which lower than the 22.92% of the routine group. In the collaborative nursing intervention measures, nursing staff were required to make rounds every 2 h after surgery, and preventive nursing measures were given for complications such as pressure ulcers and deep vein thrombosis of lower limbs, which could significantly reduce the incidence of complications. Psychological intervention in systematic nursing model could help elderly patients with femoral intertrochanteric fracture to improve their negative emotional state and reduce the incidence of adverse events. Preoperative publicity of disease-related knowledge and rehabilitation guidance manuals could make patients more actively participate in limb functional exercise and reduce the occurrence of postoperative complications. Foreign scholars have found that collaborative care model can significantly improve the postoperative quality of life of elderly patients with osteoporotic thoracolumbar compression fractures [15]. Some scholars have found that collaborative care model can effectively relieve pain and improve quality of life in patients with osteoporotic vertebral compression fractures [16]. Jiangc R L [17] found that collaborative care mode enhanced the collaboration between patients and medical service providers, shortened the length of hospital stay, and reduced postoperative pain and anxiety. The above results showed that this research scheme based on RAM model for nursing assessment identified nursing problems, established nursing goals and timely nursing intervention, and preferentially solved to the patient’s adverse psychosocial adaptation problems so that patients got a sense of adaptation and security. Nursing intervention measures such as psychological intervention of nurses and appearance of sodality of patients and patients were used to help patients reduce various stimuli, find and deal with negative emotional reactions early, and promote the correct change of patients’ cognition of the disease. Patients were encouraged to communicate with each other, and patients’ association was held regularly every week to facilitate patients to transfer effective information to each other, exchange their disease diagnosis and treatment experience and rehabilitation exercise methods, and create sufficient communication and interaction space for patients. After listening to the diagnosis and treatment experience and rehabilitation exercise methods of others, patients will think about their own problems in the whole rehabilitation process, and can adjust their wrong cognition and behavior in time, so as to obtain a better adaptation [18, 19].

The logistic multivariate regression analysis in this research showed that age, preoperative ASA classification, internal fixation method, and osteoporosis classification were risk factors that affected the recovery of hip joint function after surgery, and perioperative nursing methods were protective factors. The ROC curve analysis results showed that the internal fixation method and osteoporosis grade among the influencing factors had a certain clinical value in predicting the postoperative hip function recovery in patients with femoral intertrochanteric fractures. With the increase of age, body function, BMD and bone plasticity decrease significantly, and postoperative blood supply and nutrition cannot meet the recovery needs, resulting in a poor hip function recovery. A higher the ASA grade indicated a more severe patient’s condition and a higher the risk of anesthesia and surgery, which affected the postoperative rehabilitation effect [20]. PFNA is more conducive to recovery than other fixation methods, which can support early weight-bearing, and facilitate functional recovery. DHS is more traumatic and prone to problems such as head cutting. Osteoporosis is one of the main causes of fractures. The occurrence of osteoporosis after screw internal fixation in the treatment of fractures can affect the initial stability of fixation to cause fixation failure, and the quality of fracture healing is low after operation, leading to the worse recovery of hip joint function [21]. Perioperative collaborative nursing intervention is a protective factor for postoperative hip function recovery in patients with intertrochanteric fracture, which is consistent with the results of the previous part of this study.

In conclusion, the RAM model-based collaborative nursing method may effectively restore the hip joint function of patients with femoral intertrochanteric fracture after operation, and may contribute to reduce the perioperative pain degree of patients, improve the quality of life of patients and reduce the incidence of complications, which can be popularized and applied in clinical practice. In addition, there are many factors influencing the recovery of hip joint function in patients with femoral intertrochanteric fracture after operation, and targeted measures should be taken according to the influencing factors. Besides, it is expected to explore more suitable nursing measures for the rehabilitation of clinical fracture patients, and to strengthen the health education and rehabilitation promotion of orthopedic specialist after discharge, which are the innovation of this study. However, this study design used a retrospective analysis, which might have led to selection bias and confounding variables that could have influenced the final results. The lack of randomization might limit the internal validity of the study. In addition, due to the small sample size of the included patients, the experimental results had certain limitations in terms of universality and credibility. Therefore, a large sample, multicenter, prospective, follow-up cycle relatively long study is still needed in the future, to further objectively and accurately evaluate the clinical value of collaborative nursing based on RAM model.

## Data Availability

The datasets used and/or analyzed during the current study are available from the corresponding author on reasonable request.
